# Polymorphism of the merozoite surface protein-1 block 2 region in *Plasmodium falciparum* isolates from Mauritania

**DOI:** 10.1186/1475-2875-13-26

**Published:** 2014-01-23

**Authors:** Mohamed Salem O Ahmedou Salem, Magatte Ndiaye, Mohamed OuldAbdallahi, Khadijetou M Lekweiry, Hervé Bogreau, Lassana Konaté, Babacar Faye, Oumar Gaye, Ousmane Faye, Ali O Mohamed Salem O Boukhary

**Affiliations:** 1 Laboratoire de Biotechnologie, Faculté des Sciences et Techniques, Université des Sciences, de Technologie et de Médecine, Nouakchott, PO Box 5026, Nouakchott, Mauritanie; 2 Laboratoire d’Ecologie Vectorielle et Parasitaire, Faculté des sciences et techniques, Université Cheikh Anta Diop, Dakar, Sénégal; 3 Service de Parasitologie-Mycologie, Faculté de Médecine, Université Cheikh Anta Diop, Dakar, Sénégal; 4 Service de Parasitologie et de Mycologie, Institut National de Recherches en Santé Publique, BP 695, Nouakchott, Mauritanie; 5 Unité de Parasitologie, Département d’Infectiologie de Terrain, Institut de Recherche Biomédicale des Armées, BP 7391 223 Brétigny-sur-Orge cedex, France; 6 Aix Marseille Université, Unité de Recherche sur les Maladies Infectieuses et Tropicales Emergentes, UM 63, CNRS 7278, IRD 198, INSERM 1095, Faculté de Médecine La Timone, 27 boulevard Jean Moulin, 13385 Marseille cedex 5 Marseille, France; 7 Unité de Parasitologie Institut Pasteur, 23 Avenue Pasteur, BP 6010, 97306 Cayenne cedex Guyane, France

**Keywords:** *Plasmodium falciparum*, Malaria, Genetic diversity, Multiplicity of infection, *msp-1* gene, Mauritania

## Abstract

**Background:**

The genetic diversity of *Plasmodium falciparum* has been extensively studied in various parts of the world. However, limited data are available from Mauritania. The present study examined and compared the genetic diversity of *P. falciparum* isolates in Mauritania.

**Methods:**

*Plasmodium falciparum* isolates blood samples were collected from 113 patients attending health facilities in Nouakchott and Hodh El Gharbi regions. K1, Mad20 and RO33 allelic family of *msp-1* gene were determined by nested PCR amplification.

**Results:**

K1 family was the predominant allelic type carried alone or in association with Ro33 and Mad20 types (90%; 102/113). Out of the 113 *P. falciparum* samples, 93(82.3%) harboured more than one parasite genotype. The overall multiplicity of infection was 3.2 genotypes per infection. There was no significant correlation between multiplicity of infection and age of patients. A significant increase of multiplicity of infection was correlated with parasite densities.

**Conclusions:**

The polymorphism of *P. falciparum* populations from Mauritania was high. Infection with multiple *P. falciparum* clones was observed, as well as a high multiplicity of infection reflecting both the high endemicity level and malaria transmission in Mauritania.

## Background

Malaria remains a public health problem in Mauritania with an estimated of 250,000 clinical cases reported annually [1]. Data reported during the two last decades shown an increase in malaria cases from 26.933 in 1990 to 188.025 in 2006 [2]. The majority of infection is due to *Plasmodium falciparum,* which is responsible for more than 80% of malaria cases and entomological studies show that the main vector is *Anopheles gambiae*[1,3-7]. Throughout the country malaria is endemic with a seasonal transmission from July to October, as is the case in the most Sahelian countries [1,8]. The country can be divided into three epidemiological strata: the Saharan region in the north, representing 80% of the country’s area with annual rainfall less than 100 mm, where malaria transmission is rare. A sero-epidemiological study conducted in 1980s showed prevalence between 8 and 15% of history of infection with *P. falciparum* in this zone [9]. In the Sahelian zone, located in the southern part, between Nouakchott and Nema (annual rainfall 100-300 mm), malaria is unstable and seasonal transmission during rainy season can cause deadly epidemics [3,4]. The Sudanian region in the South, bordering with the Senegal River Valley (annual rainfall 300-600 mm), an agricultural area, including rice, where malaria transmission is stable, but with a significant difference in prevalence between the rainy season and the dry season [1]. Despite the enormous efforts that have been directed toward malaria control and prevention [10], multiple factors including insecticide resistance in anopheline vectors, the lack of effective vaccines, and the emergence and rapid spread of drug-resistant strains are the major problems for controlling and preventing malaria.

Therefore, the development of an effective malaria vaccine continues to be urgently needed. However, extensive genetic diversity in natural malaria parasite populations is a major obstacle for the development of an effective vaccine against these parasites, because antigenic diversity limits the efficiency of acquired protective immunity to malaria [11,12]. Many *P. falciparum* proteins have been proposed for use as vaccine candidate antigens, but the merozoite surface protein-1 (MSP-1) has been most studied [13,14]. Extensive genetic polymorphism of the *msp-1* gene has been identified in *P. falciparum* isolates worldwide [15-17]. It is important to investigate the diversity of *msp-1* gene, in different geographic areas for the further development of effective malaria vaccine. The gene coding the merozoite surface protein 1 (*msp-1*) has been widely used as a marker for genotyping parasite populations [18-20]. In *P. falciparum*, the surface protein MSP-1 (around 190 kDa) plays an important role in erythrocyte invasion by the merozoite [21]. The protein is a principal target of human immune responses [22] and is a promising candidate for a blood stage subunit vaccine [14,23]. The *msp-1* gene occurs as one of three distinct allelic families: K1, MAD20 and Ro33 [24]. It has 7 variable blocks that are separated either by conserved or semi-conserved regions. Block 2, a region near the N-terminal of MSP-1, is the most polymorphic part of the antigen and appears to be under the strongest diversifying selection within natural populations [21].

The genetic diversity of *P. falciparum* has been extensively studied in various parts of the world, but limited data are available from Mauritania. The only study on *P. falciparum* population genetic structure using *msp-1*, *msp-2* and *glurp* markers date back 15 years [4]. The present study examined and compared the genetic diversity of *P. falciparum* isolates among febrile patients attending health facilities in Nouakchott and Hodh El Gharbi region in Mauritania, during the peak of malaria transmission, using the polymorphic gene encoding for the merozoite surface protein-1 (MSP-1).

## Methods

### Study sites

**Figure 1:**
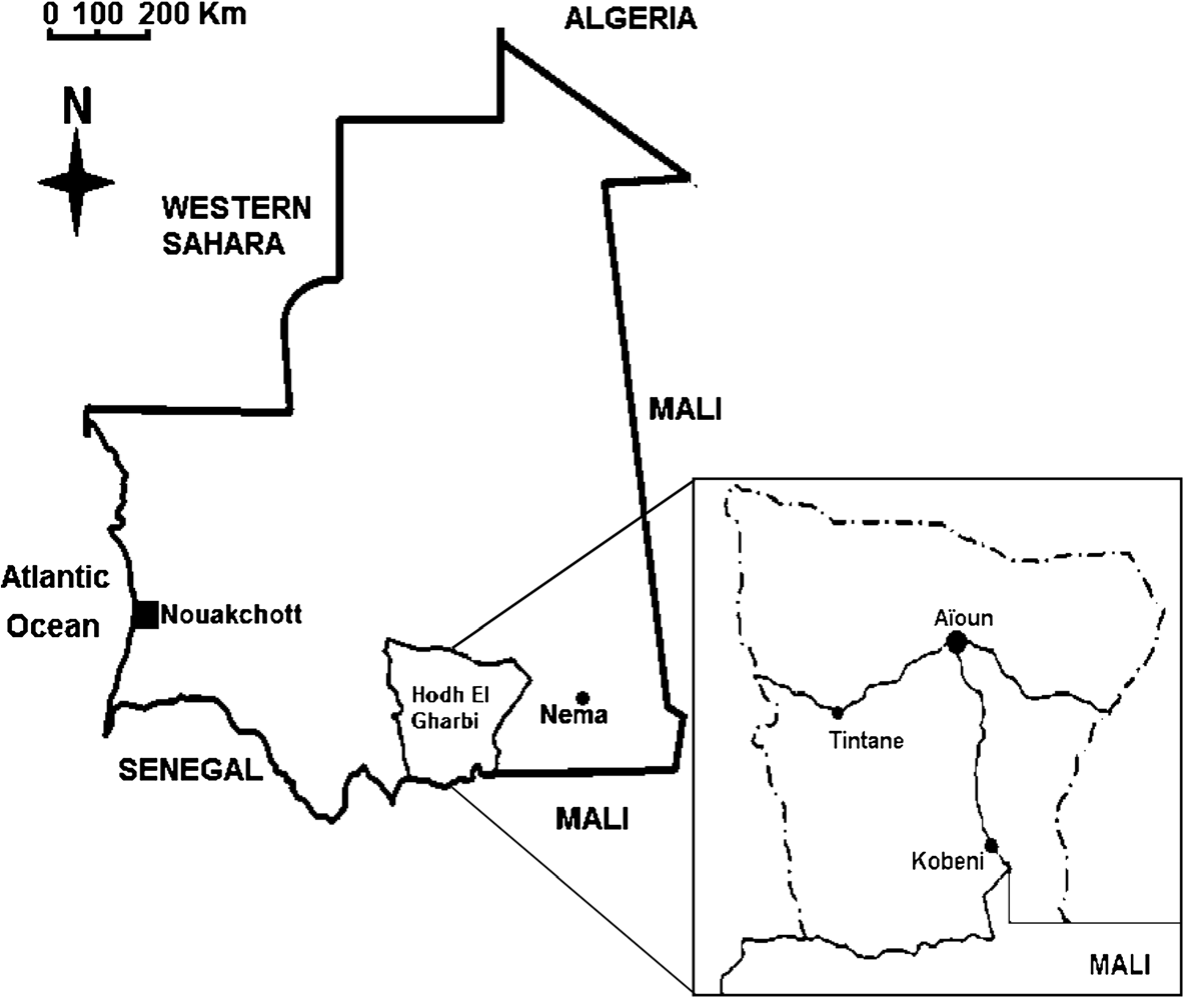
Map of Mauritania showing the study sites.

The study was conducted in Hodh El Gharbi (HG), south-east Mauritania, and in Nouakchott, the capital city of Mauritania during the rainy season in September–October 2010 (Figure 1). HG region (latitude 16° 13′ 02″ N, longitude 9° 54′ 44″ W) covers a surface area of 53,000 km^2^ with four departments, (Aïon, Kobeni, Tintane and Tamchekett). The population of HG is approximately 212,156 inhabitants [25]. Most residents are subsistence animal breeders and farmers. The climate in HG region is Sahelian characterized by a long dry season, lasting from October to June, and a rainy season extending from July to September. It receives between 200-300 mm of annual rainfall. The monthly mean temperature ranges from 24 to 37°C. A transmauritanian highway of 1,200 km from Nouakchott to Nema in the far south-eastern region of the country passes through HG from west to east. HG region can also be reached on a paved road from Nioro du Sahel in Mali. The region is poorly served by health service facilities. There is only one hospital in Aïoun and one health centre in each department. Nouakchott (18°.11′N; 16°.16′W), is situated in a Saharan zone, near the Atlantic coast at an average altitude of 7 m. It comprises nine districts and houses 743,511 inhabitants corresponding to one fourth of the whole population of the country [25]. The wet season is short, extending from July to September, with little annual variation in the amount of rainfall (50-80 mm). The monthly mean temperatures in Nouakchott varied from 21°C to 30.5°C. Nouakchott is served by five hospitals and nine health centres spread across the city. HG and Nouakchott exhibit every year high level of human migration with Nouakchott residents traveling and staying in Hodh El Gharbi during the months of July, August, September corresponding to the school vacations, and returning to Nouakchott in October and November.

### Patients and blood samples collection

The samples of this study were collected from 676 febrile patients attending Kobeni, Tintane and Aïoun health faciltities in HG region and the 'Centre Hospitalier National’, 'Centre Hospitalier Cheikh Zayed’ and 'Centre de Santé de Teyarett’ in Nouakchott. Before blood sample collection, informed consent was obtained from all participants or in case of children from their parents/legal guardians prior to their enrolment. The study was approved by 'the Direction Régionale de l’Action Sanitaire, DRAS, under the authority of the Mauritanian Ministry of Health.

During the study, if patient presented to health facilities with symptoms consistent with mild malaria, including fever, chills, headache, and a positive slide, they were offered standard ACT as first-line treatment. Among the patients enrolled, 276 (40.8%) were microscopically confirmed as *P. falciparum* infection. Out of these 114 (40.7%) patients were from Kobeni, 89(31.7%) from Tintane and 55(19.6%) from Aïoun in HG region and 18(6.5%) patients from Nouakchott. A total of 113 patients were randomly selected from the 276 *P. falciparum* positives patients by microscopy. A finger prick blood sample was taken from each consenting patient for thick and thin blood film and 2-3 drops were collected on Whatman® No. 3 filter paper, dried and stored in individual plastic bag until used for DNA extraction.

### Malaria parasite identification

Thick and thin smears were stained with Giemsa. Parasite density was determined by counting the number of asexual parasites per 200 white blood cells, and calculated per microlitre using the following formula: number of parasites x 8,000/200, assuming a white blood cell count of 8,000 cells per μl [26]. Absence of malaria parasites in 200 high power ocular fields of the thick film was considered as negative. Obtained data were graded according to parasitaemia: 1, patients with 50-499 parasites/μl; 2, patients with 500-4,999 parasites/μl; 3, patients with more than 5,000 parasites/μl.

### *Plasmodium falciparum* DNA extraction

Parasite DNA was extracted, from blood samples blotted on filter paper by methanol method described by Djimde *et al*. [27]. Briefly, 500 μl of methanol was added to a 1.5 ml microcentrifuge tube containing three or four pieces of filter paper and incubated at 15 min at room temperature. The methanol was poured off and the papers heated at 95-100°C in 50-70 μl of sterile distilled water for 15 minutes of incubation, during which the tube was gently vortexed every five minutes. The samples were centrifuged twice and the final supernatant was stored at -20°C until used for the amplification reaction. All PCR analyses were conducted in the 'Service de Parasitologie’ at the Faculty of Medicine, Université Cheikh Anta Diop, Dakar.

### PCR amplification of the *P. falciparum msp-1* gene

The highly polymorphic locus, *msp-1* block 2, was used for the genotyping of the *P. falciparum* population using the nested polymerase chain reactions (PCR) technique. The *msp -1* primers sequences used in this study are shown in Table 1[28]. For the primary amplifications (outer PCR), primer pairs corresponding to the flanking sequence of the conserved regions of *msp -1* was used (Table 1). The second amplification reactions (nested PCR) were based on the primary products using allelic-specific primers sets corresponding to K1, RO33 and Mad20 families of *msp-1*. The primers for *msp -1* used in this study are shown in Table 1. All outer and nested PCR reactions were done in 25 μl final volume. For each reaction, 200 μM each of deoxyribonucleoside triphosphate (dNTP), 1 μM of each primer, 0.5U of Taq DNA polymerase and PCR Buffer 10X containing 100 mM Tris–HCl, pH 8.3, 500 mM KCl, 15 mM MgCl2 and 0.01% gelatin were used. The outer and the nested amplification were performed on an Applied Biosystem 2720 and PTC-100 Peltier Thermal Cycler respectively using the following conditions: initial denaturation for 3 min at 94°C, followed by 30 cycles of 25 s denaturation at 94°C, 35 s annealing at 50°C, and 2 min 30 extension at 68°C. Final extension was carried out at 72°C for 3 min. Nested PCR products were visualized by electrophoresis on 2% agarose gel (Gibco-BRL) for various lengths of time depending of the predicted size of the PCR products and visualized under UV trans-illumination.

**Table 1:** **Sequences of oligonucleotide primers used to genotype *msp-1 *** **block 2 allelic types of *Plasmodium falciparum *** **isolates from Mauritania**

**Amplification/Primer**	**Sequence**	**Polymorphism**
**Outer PCR**		
Msp1F	5′AAGCCTTAGAAGATGCAGTATTGAC3′	Conserved
Msp1R	5′ATTCATTAATTTCTTCATATCCATTATC3′	
**Nested PCR**		
K1F	5′AAGAAATTACTACAAAAGGTG3′	K1 family specific
K1R	5′TGCATCAGCTGGAGGGCTTGCACCAC3′	K1 family specific
Ro33F	5′AGGATTTGCAGCACCTGGAGATCT3′	Ro33 family specific
Ro33R	5′GAGCAAATACTCAAGTTGTTGCA3′	Ro33 family specific
Mad20F	5′TGAATTATCTGAAGGATTTGTACGTC3′	Mad20 family specific
Mad20R	5′GAACAAGTCGAACAGCTGTTA3′	Mad20 family specific

### Allelic distribution and multiplicity of infection

The *msp-1* alleles were categorized by their molecular weights and considered the same if their molecular weights were approximately within 10 bp [29]. The multiplicity of infection (MOI), or complexity of infection, was estimated by the average number of PCR fragments per infected individual, as described by Zwetyenga *et al.*[30].

### Statistical analyses

Statistical analyses were performed using MedCalc for Windows, version 12.3.0.0 (MedCalc Software, Mariakerke, Belgium). Proportions were compared for significance using the χ^2^-test. Spearman’s rank correlation coefficients were calculated to assess associations between multiplicity of infection, parasite densities and age groups of patients under study. A *P* value of ≤ 0.05 was considered indicative of a statistically significant difference.

## Results

### Characteristics of the study population

Out of the 113 randomly selected *P. falciparum* patients attending health facilities in study sites, 31.8% (36/113) were from Kobeni, 34.5% (39/113) from Tintane, 17.7% (20/113) from Aïoun and 16% (18/113) from Nouakchott (Table 2). Males (70/113; 61.9%) were significantly higher (χ^2^ = 6.4; *p* = 0.01) than females (43/113; 38.1%). Patients belonging to the age group 5-19 years were the most represented with 55.8% (63/113) followed by those of 20-39 years of ages with 28.3% (32/113), patients whose ages ≥ 40 years with 12.4% (14/113) and children under five years representing 3.5% (4/113).

**Table 2:** **Population characteristics of *P. falciparum *** **infected patients from different study sites in Mauritania**

**Characteristics**	**Kobeni**	**Tintane**	**Aïoun**	**Nouakchott**	**Total**
	**n (%)**	**n (%)**	**n (%)**	**n (%)**	**n (%)**
***Gender***					
Male	20(55.6)	27(69.2)	12(6)	11(61.1)	70(61.9)
Female	16(44.4)	12(30.8)	8(4)	7(38.9)	43(38.1)
***Age group***					
< 5	1(2.8)	1(2.56)	2(10.0)	0	4(3.5)
5-19	21(58.3)	23(59.0)	13(65.0)	6(33.3)	63(55.8)
20-39	13(36.1)	7(17.95)	4(20.0)	8(44.5)	32(28.3)
≥ 40	1(2.8)	8(20.5)	1(5.0)	4(22.2)	14(12.4)
Total	36(31.8)	39(34.5)	20(17.7)	18(16.0)	113(100)

n: number of individuals.

### Allelic polymorphism of *msp-1*

Allele typing revealed highly polymorphic nature of Mauritanian *P. falciparum* isolates with respect to *msp-1* gene. K1, Mad20 and Ro33 MSP1 allele’s types were identified. The total number of different sized alleles detected in this sample was 27. Among them, nine for K1 (160-400 bp), 12 for Mad20 (140-400 bp) and six for Ro33 (160-360 bp) allele families were noted. Frequencies of different *msp-1* alleles and their combinations and multiplicity of infection are shown in Table 3. The frequency of samples with only K1, Mad20 and Ro33 were 10.6% (12/113), 2.6% (3/113) and 4,4% (5/113) respectively. K1/Mad20, K1/Ro33 and Mad20/Ro33 combinations were found in 21.2% (24/113), 16.8% (19/113) and 2.6% (3/113) of samples respectively and 41.6% (47/113) of them harbored all three allelic types. Prevalence of K1, Mad20 and Ro33 allelic types was 90.0% (102/113), 68.1% (76/113) and 65.5% (74/252) respectively. Multiple clones infections over all study sites was 82.3% (93/113) with a minimum of 72.2% (13/18) in Nouakchott and a maximum of 90.0% (18/20) in Aïoun. Multiplicity of infection (MOI) was highest in *P. falciparum* infected patients enrolled in Aïoun health facility (4.3 genotypes per infection) and lowest in those from Nouakchott health facilities (2.3; mean of three health facilities). However, obtained MOI values were not statistically different from each other (*p = 0.56*). The estimated MOI of all studied area was 3.2 genotypes per infection.

**Table 3:** ***Msp-1 *** **block 2 allelic type frequencies and multiplicity of infection of *P. falciparum *** **isolates from different study sites in Mauritania**

**Allelic type**	**Kobeni**	**Tintane**	**Aïoun**	**Nouakchott**	**Total**
	**n (%)**	**n (%)**	**n (%)**	**n (%)**	**n (%)**
K1	4(11.1)	6(15.4)	1(5.0)	1(5.5)	12(10.6)
Mad20	1(2.8)	0	1(5.0)	1(5.5)	3(2.6)
Ro33	0	2(5.1)	0	3(16.7)	5(4.4)
K1/Mad20	7(19.4)	10(25.6)	6(30.0)	1(5.5)	24(21.2)
K1/Ro33	9(25.0)	4(10.2)	0	6(32.3)	19(16.8)
Mad20/Ro33	2(5.5)	0	0	1(5.5)	3(2.6)
K1/Mad20/Ro33	13(36.1)	17(43.6)	12(60.0)	5(27.8)	47(41.6)
Total	36(100.0)	39(100.0)	20(100.0)	18(100.0)	113(100.0)
Total K1	33(91.6)	37(94.9)	19(90.0)	13(72.2)	102(90.0)
Total Mad20	23(63.8)	27(69.2)	19(90.0)	7(38.8)	77(68.1)
Total Ro33	24(66.6)	23(58.9)	12(60.0)	15(83.3)	74(65.5)
Multiclonal isolates	31(86.1)	31(79.5)	18(90.0)	13(72.2)	93(82.3)
MOI	3.1	3.2	4.3	2.3	3.2

n: number of individuals; MOI: multiplicity of infection.

### Relationship between multiplicity of infection, age groups and parasite densities

Age group distribution of *P. falciparum*-infected patients and *msp-1* block 2 allelic types is shown in Table 4. The prevalence of K1, Mad20, Ro33 and their different combinations was significantly higher among patient aged 5-19 years compared to other age groups (*p < 0.05*) (Table 4). However, no significant correlation between multiplicity of infections and age groups of patients (Spearman rank coefficient = 0.2; *p = 0.8*) was noted. The distribution of K1, Mad20 and Ro33 according to parasitaemia is shown in Table 5. This study found that the individual distribution of K1, Mad20, Ro33 was not significantly different among parasitaemic groups (p = 0.94). Multiclonal isolates was not significantly different neither between 50-499 parasites/μl and ≥ 5000 parasites/μl (*p = 0.09*), or between 50-499 parasites/μl and 500-4999 parasites/μl (p = 0.08). However, estimated parasitaemia and multiplicity of *P. falciparum* infection was significantly correlated (Spearman rank coefficient = 1; *p* < 0.01).

**Table 4:** **Distribution of *P. falciparum *** **msp-1 block 2 allelic type among age groups of *P. falciparum *** **infected patients from Mauritania**

	**Age group (years)**				
**Allelic type**	**< 5**	**5-19**	**20-39**	**≥40**	**Total**
	**n (%)**	**n (%)**	**n (%)**	**n (%)**	**n (%)**
K1	1(0.9)	8(7.1)	1(0.9)	2(1.8)	12(10.6)
Mad20	0	1(0.9)	2(1.8)	0	3(2.6)
Ro33	0	2(1.8)	2(1.8)	1(0.9)	5(4.4)
K1/Mad20	1(0.9)	17(15.0)	6(5.3)	0	24(23.0)
K1/Ro33	1(0.9)	10(8.8)	6(5.3)	2(1.8)	19(16.8)
Mad20/Ro33	0	2(1.8)	1(0.9)	0	3(2.6)
K1/Mad20/Ro33	1(0.9)	23(20.3)	14(12.4)	9(7.9)	47(41.6)
Total	4(3.5)	63(55.7)	32(28.4)	14(12.4)	113(100.0)
Total K1	4(100.0)	58(92.1)	27(84.3)	13(92.8)	102(90.3)
Total Mad20	2(50.0)	43(68.2)	23(71.8)	9(64.3)	77(68.1)
Total Ro33	2(50.0)	37(58.7)	23(71.8)	12(85.7)	74(65.5)
Multiclonal isolates	3(2.6)	52(46.0)	27(23.9)	11(9.7)	93(82.3)
MOI	3.25	3.21	3.03	3.37	3.2

n: number of individuals; MOI: multiplicity of infection.

**Table 5:** **Distribution of Msp-1 block 2 allelic types among parasitaemic groups of *P. falciparum *** **infected patients from Mauritania**

**Allelic type**		Parasitaemia*		
	**50-499 n (%)**	**500-4999 n (%)**	**≥5000 n (%)**	**Total**
K1	1(0.9)	6(5.3)	5(4.4)	12(10.6)
Mad20	1(0.9)	2(1.8)	0	3(3.5)
Ro33	0	2(1.8)	3(2.6)	5(4.4)
K1/Mad20	1(0.9)	10(8.8)	13(11.5)	24(21.2)
K1/Ro33	6(5.3)	9(7.9)	4(3.5)	19(16.8)
Mad20/Ro33	0	1(0.9)	2(1.8)	3(2.6)
K1/Mad20/Ro33	2(1.8)	22(19.5)	23(20.3)	47(41.6)
Total	11(9.7)	52(46.0)	50(44.3)	113(100.0)
Total K1	10(90.9)	47(90.4)	45(90.0)	102(90.3)
Total Mad20	4(36.4)	35(67.3)	38(76.0)	77(68.1)
Total Ro33	8(72.7)	34(65.4)	42(84.0)	74(65.5)
Multiclonal isolates	9(7.9)	42(37.2)	42(37.2)	93(82.3)
MOI	2.6	3.4	3.6	3.2

(*) parasite/μl of blood; n: number of individuals; MOI: multiplicity of infection.

## Discussion

This study was undertaken to assess the polymorphism of the *merozoite surface protein-1* block 2 allelic types in *P. falciparum* isolates from different study sites in Mauritania. *Plasmodium falciparum* is thought to be responsible of nearly 80% of diagnosed malaria cases in the country [1]. A better understanding of population structure of *P. falciparum* genotypes may be an important element for implementing malaria control strategies in the country. Data showed a relatively high polymorphic nature of K1, Mad20 and Ro33 *msp-1* allelic types according to the number of band sizes (27 different PCR products: 9 K1, 12 Mad20 and 6 Ro33) among Mauritanian *P. falciparum* isolates. Comparable results were found by Jordan et al. [4]; authors noted 11, 8 and 2 different sized alleles respectively for K1, Mad20 and Ro33 in *P. faliparum* isolates from malaria patients in Aïoun and Kobeni area located in southern Mauritania. The number of different *msp-1* alleles observed among *P. falciparum* isolates is also comparable to that founded by Konate *et al.*[31] in the holoendemic area of Dielmo (Senegal) where 33 *msp-1* alleles were evidenced, but less than that reported by Soulama *et al.*[29] who founded 41 different sized alleles for *msp-1* block 2 region in *P. falciparum* isolates collected from children with uncomplicated malaria infection living in Burkina Faso. The study also revealed the predominance of K1 type alleles either at study site level or throughout the parasite population compared to Mad20 and Ro33. Similar finding have previously reported among *P. falciparum* isolates from Aïoun and Kobeni [4]. In Dakar, *P. falciparum* isolates from 48 Senegalese patients hospitalized for malaria showed a prevalence of 68% for K1 alleles [32]. Most of the isolates were mixed infections (82.3%) which harbored more than one allele type. However, prevalence of patients with multiclonal infection was similar in all study sites. A relatively high multiplicity of infections (MOI) was observed among *P. falciparum* patients either at study site level (2.33-4.25) or over all the study sites (3.22) reflecting the high intensity level of malaria transmission during the study period. When they compared 63 *P. falciparum* positve samples taken from febrile patients in Aïoun and 110 positive samples from Kobeni, Jordan *et al.*[4] reported a multiple infections of 1.57 and 2.34, respectively. It is reported that the multiplicity of infection in an infected patient may be due to an important entomological inoculation rate as shown in the Senegal village of Dielmo where patients receive a large number of infective bites [31], or by a single mosquito carrying several parasitic clones in his inoculums [33]. In the context of the present study, it is difficult to speculate on the influence of mosquito inoculum on the multiplicity of infection because no such data is available for the study sites in Mauritania. The MOI values reported in this study are consistent with those reported from regions with high malaria endemicities [34]. Furthermore, there was no association between MOI and age of patients. Investigations conducted in Senegal (Ndiop village where malaria is mesoendemic and seasonal) and Benin (Cotonou), founded that MOI was not affected by age of malaria patients [31,35]. Previous studies regarding the variation of MOI over age have suggested that the influence of age on the multiplicity of infection is highly affected by endemicity of malaria which is probably a reflection of the development of anti-parasite specific immunity [36]. However, this finding contrasting with reports from other locations [29,31,37]. A positive correlation between multiplicity of infection and parasite densities was observed in this study (MOI = 2.6-3.6). This is consistent with many reports demonstrating that higher parasite densities increase the probability of detecting concurrent clones in an individual [18,38]. This study used *P. falciparum* isolates from patients with clinical symptoms, so that the MOI may be lower than that reported in asymptomatic patients [39]. Many reports showed that MOI would reflect that infected individual has immunity against malaria [31]. Thus, the MOI should be studied in asymptomatic *P. falciparum*-infected population in Mauritania further, particularly in children. The similarity between *P. falciparum* strains could be explained by population migration between study sites that may allow for exchanges of parasite population. Similar results were noted in many malaria endemic areas [40-42]. The present study is the second report based on molecular epidemiology of *P. falciparum* following the work conducted by Jordan *et al.*[4] in Aïoun and Kobeni Department of HG region and the first with respect to Tintane department in the same region and Nouakchott, the capital city of Mauritania. It demonstrates that *P. falciparum* populations, at leat in Kobeni and Aïon, had maintained a high level of polymorphism in the *msp-1* block 2 region. Studies using a larger number of blood samples collected from different geographic areas in Mauritania are required not only to determine the nationwide parasite heterogeneity and detailed malaria epidemiology but also to implement malarial control programmes in this neglected malaria country.

## Conclusions

*Plasmodium falciparum* isolates from Mauritania exhibited a high degree of genetic polymorphism in *msp-1* gene and most of the infected patients carried multiple clones of parasites reflecting the high level of malaria endemicity in study sites during malaria transmission season.

## Competing interests

The authors declare that they have no competing interests.

## Authors’ contributions

MSOAS conceived of the study, carried out molecular study and performed statistical analysis; MN, BF, OG conceived and supervised molecular analyses and participated in the paper drafting; KML, OAM, carried out the microscopic analysis and participated in statistical analyses; HB, LK, OF, AOMSB conceived of the study, participated in its design and helped to draft and critically analysed the manuscript. All authors read and approved the final manuscript.
